# Prognostic Impact of Glomerular Filtration Rate Decline on Survival Outcomes in Metastatic Renal Cell Carcinoma Treated with Targeted Therapy

**DOI:** 10.3390/medicina61091574

**Published:** 2025-08-31

**Authors:** Oktay Halit Aktepe, Ahmet Melih Arslan, Ozge Yetginoglu, Hatice Altas, Canberk Sencan, Mehmet Sinan Akarca, Hasan Cagri Yildirim, Huseyin Salih Semiz, Ilkay Tugba Unek, Aziz Karaoglu, Mustafa Erman, Suayib Yalcin

**Affiliations:** 1 Department of Medical Oncology, Dokuz Eylul University, Izmir 35330, Turkey; 2 Department of Internal Medicine, Dokuz Eylul University, Izmir 35330, Turkey; 3 Department of Medical Oncology, Ege University, Izmir 35100, Turkey; 4 Department of Medical Oncology, Hacettepe University Cancer Institute, Ankara 06230, Turkey

**Keywords:** glomerular filtration rate, metastatic renal cell carcinoma, prognosis, targeted therapy

## Abstract

*Background and Objectives:* The prognostic significance of dynamic changes in glomerular filtration rate (GFR) during targeted therapies in metastatic renal cell carcinoma (mRCC) is not well understood. Thus, we aimed to investigate the prognostic significance of GFR value at 6 months in patients with mRCC receiving first-line targeted therapy. *Materials and Methods:* This retrospective cohort study included 260 mRCC patients at two tertiary centers in Turkey between 2015 and 2025. Patients were stratified into three groups according to GFR at 6 months: ≥60, 30–60, and <30 mL/min/1.73 m^2^. Kaplan–Meier curves were used to estimate progression-free survival (PFS) and overall survival (OS) in prognostic subgroups. Cox proportional hazard models assessed associations between clinicopathologic variables, including GFR categories, and PFS and OS. *Results:* The median PFS for the cohort was 11.1 months (95% confidence interval [CI]: 9.3–12.9), and the median OS was 40.0 months (95% CI: 30.3–49.7). In multivariate analysis, GFR < 30 mL/min/1.73 m^2^ was independently associated with shorter PFS (hazard ratio [HR]: 1.54, 95% CI: 1.01–2.33, *p* = 0.040) and OS (HR: 3.80, 95% CI: 2.06–7.01, *p* < 0.001), while GFR 30–60 mL/min/1.73 m^2^ was linked to reduced OS (HR: 2.07, 95% CI: 1.08–3.98, *p* = 0.028). Additional independent predictors of worsened PFS were intermediate (*p* = 0.028) and poor IMDC risk (*p* < 0.001. For OS, liver metastases (*p* = 0.017), bone metastases (*p* = 0.014), brain metastases (*p* = 0.002), and intermediate (*p* = 0.014) or poor IMDC risk (*p* < 0.001) were also significant. *Conclusions:* In patients with mRCC treated with targeted therapy, the GFR at 6 months is an independent factor in predicting survival outcomes, indicating the clinical significance of serial kidney function monitoring.

## 1. Introduction

Metastatic renal cell carcinoma (mRCC) remains a challenging problem in oncology worldwide and is responsible for a significant portion of cancer-related deaths [1], despite advancements in treatment over the last two decades, including immune checkpoint inhibitors (ICIs) and vascular endothelial growth factor receptor (VEGFR) inhibitors [2,3]. The International Metastatic RCC Database Consortium (IMDC) has been used for risk stratification and treatment choices [4]. However, there is an urgent need to integrate novel biomarkers into existing prognostic systems to guide better prognostication and personalized treatment selection.

Glomerular filtration rate (GFR), a key hallmark of kidney function, is associated with cardiovascular and all-cause mortality in the general population and individuals with chronic kidney disease (CKD) [5,6]. Impaired GFR is increasingly being investigated not only as a sign of toxicity but also as a predictive marker of survival outcomes in several types of cancers, including mRCC [7,8,9,10]. Prior studies have primarily focused on baseline renal function as a predictor of treatment toxicity and prognosis in mRCC patients receiving VEGFR-targeted therapies [10,11,12]. However, limited data exist on the prognostic significance of GFR decline throughout targeted therapy. Therefore, we conducted a retrospective analysis to investigate the association between changes in renal function, specifically GFR decline at 6 months, in mRCC patients treated with first-line targeted therapy. In the present study, we hypothesized that a significant decline in GFR over the initial treatment period would contribute to the growing body of evidence, providing better risk classification and personalized treatment strategies for mRCC.

## 2. Materials and Methods

### 2.1. Patient Population

This retrospective cohort study was conducted at Dokuz Eylul University and Hacettepe University, two tertiary cancer centers in Turkey, evaluating the prognostic importance of GFR at 6 months in patients diagnosed with mRCC who received first-line targeted therapy between January 2015 and January 2025. We collected clinical and pathological data from each center’s electronic medical records and oncology databases. Baseline demographic and clinicopathologic characteristics included age at therapy initiation, body mass index (BMI), sex, tumor grade, histological subtype (clear cell, papillary, chromophobe, or unclassified), and sites of metastases (lung, bone, liver, brain, lymph nodes, or other). Systemic treatment history was documented, including type of first-line VEGFR-targeted agent (sunitinib, pazopanib, or cabozantinib) and subsequent lines of therapy.

For each patient, the IMDC risk score was calculated using baseline Karnofsky performance status, hemoglobin, corrected calcium, neutrophil count, platelet count, and time from diagnosis to initiation of systemic therapy. According to this system, patients were categorized into three risk groups: favorable, intermediate, and poor risk. Patients were included based on: (1) age ≥ 18 years, (2) available baseline and 6-month serum creatinine measurements to calculate estimated GFR, (3) baseline GFR ≥ 30 mL/min/1.73 m^2^, (4) histologically confirmed mRCC, (5) treated with first-line targeted therapy, including sunitinib, pazopanib, or cabozantinib as monotherapy, and (6) no disease progression in the first 6 months after treatment with targeted therapy. Patients were excluded if they had received ICIs as part of their initial treatment, underwent nephrectomy after treatment initiation, or had incomplete clinical data or a baseline GFR < 30. Baseline GFR values were recorded within 2 weeks prior to initiation of targeted therapy, and follow-up values were obtained at 6 months (±2 weeks). Estimated GFR was calculated from serum creatinine values using the CKD-EPI equation. Patients were categorized into GFR categories of >60, 30–60, and <30 mL/min/1.73 m^2^ at 6 months according to the Kidney Disease: Improving Global Outcomes (KDIGO) guidelines [13].

### 2.2. Statistical Analysis

Continuous variables are expressed as medians with interquartile ranges (IQRs) when not normally distributed, and mean ± standard deviation (SD) when normally distributed. Categorical variables are presented as frequencies and percentages. Group comparisons in continuous and categorical variables were performed with the Mann–Whitney U test and the chi-squared test, respectively. One-way analysis of variance (ANOVA) was used to determine the differences among multiple independent groups. Progression-free survival (PFS) was defined as the time frame between first-line targeted therapy and clinical and radiologic disease progression or death. Overall survival (OS) was defined as the time frame between initiation of first-line targeted therapy and death from any cause or last follow-up. The Kaplan–Meier method was used for the estimation of survival outcomes, and differences between prognostic subgroups were assessed using the log-rank test. Univariate Cox proportional hazard regression analysis was conducted to explore associations between clinicopathologic variables and survival outcomes. Variables with a *p*-value ≤ 0.20 in univariate analysis were evaluated in our multivariate model. Multivariate Cox regression was performed to explore the independent variables in predicting survival outcomes. All statistical analyses were performed using SPSS Statistics version 27 (IBM Corp., Armonk, NY, USA). A two-sided *p*-value < 0.05 was considered statistically significant for all analyses.

## 3. Results

### 3.1. Patient Characteristics and Study Design

Baseline clinicopathologic characteristics of the whole cohort and GFR subgroups are presented in Table 1. A total of 260 patients with mRCC who met the inclusion criteria were included. The mean age at treatment initiation was 60.3 years (SD: 10.6), and 72.7% were male. Clear cell histology accounted for the most cases (76.5%), and 65.8% of patients had grade III–IV tumors. Across the non-clear histological subtypes, papillary RCC was the most common subtype (*n* = 38, 62.3%), followed by chromophobe RCC (*n* = 15, 24.6%) and unclassified types (*n* = 8, 13.1%). At baseline evaluation, pulmonary metastases were the most common metastatic site (73.1%), followed by bone (27.7%), liver (19.6%), and brain metastases (6.9%). There were no significant differences among GFR groups regarding age, sex, BMI, histology, or tumor grade. However, the presence of lung metastasis (*p* = 0.040) and IMDC risk category distribution (*p* = 0.014) differed significantly between GFR groups. Patients with GFR < 30 mL/min/1.73 m^2^ were more often classified in the favorable IMDC risk group (20.5%) compared with those in higher GFR categories, whereas individuals with a GFR ≥ 60 mL/min/1.73 m^2^ showed the greatest proportion of poor-risk cases (36.7%). Regarding systemic therapy, pazopanib was the most common first-line agent used (54.2%), followed by sunitinib (35.7%) and cabozantinib (10.1%), with no significant difference in distribution across GFR groups (*p* = 0.410). In the second-line setting, nivolumab (48.2%) and axitinib (25.0%) were the most frequently administered therapies, while in the third-line setting, axitinib was the most common treatment choice (59.3%).

### 3.2. Survival Outcomes

**Figure 1:**
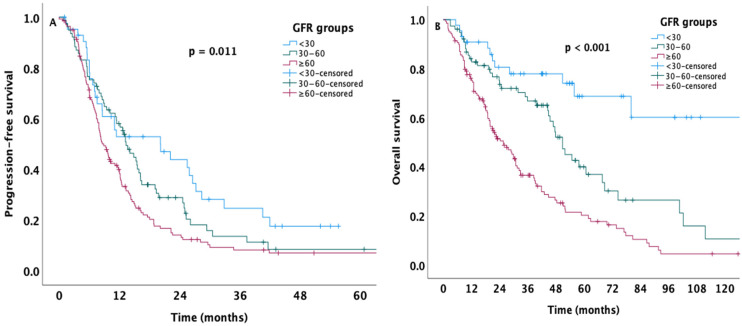
Kaplan–Meier curves demonstrating PFS (**A**) and OS (**B**) of the patients stratified according to GFR values at 6 months after initiation.

The median PFS for the overall cohort was 11.1 months (95% confidence interval [CI]: 9.3–12.9). When stratified by baseline GFR, a stepwise reduction in PFS was observed with declining renal function (*p* = 0.011, Figure 1A). Patients with GFR ≥ 60 mL/min/1.73 m^2^ achieved the longest median PFS of 20.2 months (95% CI: 7.7–32.6), followed by those with GFR 30–60 mL/min/1.73 m^2^ (13.3 months (95% CI: 10.7–16). Patients with GFR < 30 mL/min/1.73 m^2^ had the shortest PFS, with a median of 8.5 months (95% CI: 6.9–10.1 Across all patients, the median OS was 40.0 months (95% CI: 30.3–49.7). Kaplan–Meier analysis showed a significant difference in OS across GFR subgroups (*p* < 0.001, Figure 1B). Patients with preserved renal function (GFR ≥ 60 mL/min/1.73 m^2^) achieved the most extended survival, with a mean OS of 119.4 months (95% CI: 93.5–145.3, median not reached). However, the intermediate GFR group (30–60 mL/min/1.73 m^2^) had a median OS of 51.0 months (95% CI: 45.1–56.8), whereas patients with impaired renal function (GFR < 30 mL/min/1.73 m^2^) had the shortest survival, with a median OS of 25.7 months (95% CI: 19.3–32.0). Patients in the favorable-risk group had the most prolonged survival, with a median OS of 78 months (95% CI: 24.1–131.8). However, the median OS times of intermediate- and poor-risk groups were 48 months (95% CI: 38.4–57.5) and 13 months (95% CI: 8.7–17.2), respectively.

As shown in Table 2, univariate Cox regression analysis showed that poor IMDC risk (hazard ratio [HR]: 4.36, 95% CI: 2.64–7.20, *p* < 0.001), intermediate IMDC risk (HR: 1.70, 95% CI: 1.08–2.68, *p* = 0.022), and GFR < 30 (HR: 1.74, 95% CI: 1.16–2.62, *p* = 0.007) were significantly associated with inferior PFS. However, the potential variables in predicting PFS were sex (HR: 0.79, 95% CI: 0.58-1.08 *p* = 0.149), lung metastasis (HR: 0.78, 95% CI: 0.57–1.07, *p* = 0.126), liver metastasis (HR: 1.39, 95% CI: 0.98-1.97, *p* = 0.062), and bone metastasis (HR: 1.32, 95% CI: 0.98–1.78, *p* = 0.063). As shown in Table 3, in the multivariate model conducted with significant and potential variables, GFR < 30 remained independently associated with shorter PFS (HR: 1.54, 95% CI: 1.01–2.33, *p* = 0.040), along with intermediate (HR: 1.67, 95% CI: 1.05–2.66, *p* = 0.028) and poor IMDC risk (HR: 4.13, 95% CI: 2.47–6.91, *p* < 0.001). Regarding OS, GFR < 30 (HR: 4.79, 95% CI: 2.62–8.73, *p* < 0.001) and GFR 30–60 (HR: 2.45, 95% CI: 1.29–4.66, *p* = 0.006) were significantly associated with shorter OS compared to GFR ≥ 60 (Table 2). In addition to GFR, the other significant variables in predicting worsened OS were higher tumor grade (HR: 1.45, 95% CI: 1.03–2.02, *p* = 0.030), liver metastasis (HR: 1.47, 95% CI: 1.01–2.13, *p* = 0.043), bone metastasis (HR: 1.66, 95% CI: 1.19–2.32, *p* = 0.004), brain metastasis (HR: 1.88, 95% CI: 1.10–3.22, *p* = 0.020), and intermediate (HR: 2.38, 95% CI: 1.29-4.40, *p* = 0.005) and poor IMDC risk (HR: 6.32, 95% CI: 3.32–12.03, *p* < 0.001). Non-clear cell histology was determined as a potential variable in predicting OS (HR: 1.27, 95% CI: 0.88–1.82, *p* = 0.195). As shown in Table 3, in the multivariate model conducted with significant and potential variables, GFR < 30 (HR: 3.80, 95% CI: 2.06–7.01, *p* < 0.001) and GFR 30–60 (HR: 2.07, 95% CI: 1.08–3.98, *p* = 0.028) remained independent predictors of inferior OS. Other independent variables in predicting OS were liver metastasis (HR: 1.60, 95% CI: 1.09-2.37, *p* = 0.017), bone metastasis (HR: 1.54, 95% CI: 1.09–2.18, *p* = 0.014), brain metastasis (HR: 2.44, 95% CI: 1.38–4.33, *p* = 0.002), and intermediate (HR: 2.13, 95% CI: 1.16–3.90, *p* = 0.014) and poor IMDC risk (HR: 5.81, 95% CI: 3.07–10.99, *p* < 0.001).

## 4. Discussion

Our study reveals that a significant decline in GFR at 6 months after the initiation of targeted therapy is independently associated with OS in patients with mRCC. These results demonstrate the prognostic value of dynamic renal function monitoring beyond baseline evaluations, indicating that changes in renal physiology over time may reflect both medication toxicity and the biology of the underlying disease. Although targeted therapies have been the standard of care in the mRCC field for many years, the immunotherapy revolution has significantly reduced the use of these agents. However, despite this advancement, targeted therapy agents serve as key components in combination regimens with ICIs in first-line settings and remain vital in later-line treatment after progression on immunotherapy-based combinations, indicating their critical roles in the comprehensive management of mRCC.

Inhibition of VEGFR signaling results in loss of fenestrations, endothelial cell apoptosis, and podocyte injury by disrupting the maintenance of the glomerular filtration barrier [14,15]. Clinically, this often manifests as proteinuria, a clear sign of glomerular dysfunction that accompanies or precedes a decline in GFR. Additionally, chronic VEGF blockade can induce basement membrane duplication, glomerular capillary thrombosis, and endothelial swelling, which are the hallmarks of thrombotic microangiopathy [14,15,16,17]. While overt TMA is rare, patients receiving long-term targeted therapy may experience increasing nephron loss and irreversible deterioration in renal function as a result of subclinical endothelial dysfunction [18]. Hypertension, a well-known on-target consequence of VEGFR inhibition, contributes to the pathophysiology of renal impairment in patients receiving targeted therapy [19]. Glomerulosclerosis can be accelerated by elevated systemic vascular resistance and glomerular hypertension, especially in patients who already have CKD or concomitant diseases like diabetes or cardiovascular disease [20]. Taking these data into consideration, a “double hit” on the renal microvasculature may result from the interaction of drug-induced hypertension and endothelial dysfunction, particularly over extended treatment durations.

There are several plausible biological explanations for why a decline in GFR may predict worse OS in mRCC patients treated with targeted therapy. One possibility is that therapy-induced CKD may drive systemic inflammation, metabolic disturbances, and cardiovascular complications, all of which have been shown to have a detrimental impact on survival [21,22]. Second, patients developing renal impairment during treatment often require dose reductions or interruptions [11], potentially decreasing therapeutic efficacy. Third, decreasing GFR may indicate an accumulation of subclinical vascular or tubular damage and reduced physiological reserve [23], which could limit the patient’s ability to tolerate further treatment.

Initial fluctuations in kidney function during targeted therapy are often transient and may be caused by acute hemodynamic changes, hydration status, or reversible drug effects. These are not reliable indicators of permanent kidney damage. Khan et al. demonstrated that the median time to maximum renal insufficiency during mRCC treatment with targeted therapy was 6.6 months [24]. Therefore, we selected a 6-month interval to avoid the effects of short-term, non-specific fluctuations and to better capture permanent, biologically significant changes in kidney function. Macfarlane et al. found that CKD had no detrimental effect on response rates or OS in patients treated with VEGFR-targeted therapies [12]. Similarly, Masini et al. observed that impaired kidney function did not lessen the efficacy of first-line pazopanib in mRCC patients [25]. Nonetheless, these observations appear to contrast with broader oncological evidence suggesting that CKD contributes to higher cancer-specific mortality, likely due to immune suppression, oxidative stress, and chronic inflammation [26,27]. Izzedine et al. demonstrated that treatment with VEGFR inhibitors could induce both acute and chronic renal injury, requiring dose modifications [28]. Moreover, Porta et al. have highlighted the importance of identifying renal toxicity during anti-angiogenic therapy [20]. Beyond RCC, reduced GFR has been linked to worse prognosis in hematological malignancies, such as diffuse large B-cell lymphoma [8], and in solid tumors, where reduced estimated GFR was associated with higher cancer-specific mortality [7]. Similarly to these findings, our findings support the incorporation of renal function assessments during targeted therapy into standard mRCC follow-up protocols by highlighting that post-treatment decline in GFR at 6 months could serve as an independent determinant in predicting PFS and OS in patients with mRCC.

These results have significant clinical implications. Incorporating routine renal function monitoring into the long-term care of patients undergoing targeted therapy is essential for safety, prognostic considerations, and treatment planning. Clinicians should consider prompt nephrology referral, more stringent blood pressure management, and volume status optimization for mRCC patients receiving targeted therapy who have had early or persistent reductions in GFR. In some cases, it can be acceptable to switch to different regimens, including ICIs, that have a better renal safety profile.

The present study has some limitations. First, its retrospective design limits causal inference, and residual confounding effects cannot be ruled out despite multivariable adjustment. Second, we did not assess proteinuria, urinary biomarkers, or changes in kidney imaging, which could provide additional mechanical insights. Third, GFR measurements were evaluated at baseline and at a fixed six-month interval, which may not capture the full dynamic changes of renal function during therapy. Patterns such as gradual decline, stabilization, or recovery may provide additional prognostic information, but these could not be comprehensively assessed due to the retrospective nature of our dataset. Future prospective studies with serial GFR measurements at multiple time points will be essential to validate and extend our findings. Fourth, our study population was limited to two tertiary centers within a single country, which may constrain the generalizability of our findings to more diverse patient populations. However, we attempted to minimize potential bias by including a relatively large sample *(n* = 260) from two independent high-volume cancer centers and by performing multivariate analyses adjusting for known prognostic factors (e.g., IMDC risk groups, metastatic sites, tumor grade). Additionally, all patients in our cohort received targeted therapy as monotherapy in first-line setting. Future research should evaluate whether the prognostic impact of GFR decline persists in patients receiving contemporary targeted therapy–ICI combinations. Finally, the impact of genomic and transcriptomic heterogeneity, which were not consistently available in our dataset, and the absence of generally accepted confounding characteristics make it difficult to define an ideal cohort in mRCC.

## 5. Conclusions

The present study demonstrated that GFR decline at 6 months, but not baseline GFR, is associated with worsened PFS and OS in mRCC treated with first-line targeted therapy. This finding underscores the importance of monitoring kidney function over time for prognostic purposes and suggests that changes in GFR during treatment may reflect a combination of treatment-related toxicity, underlying comorbidities, and the intrinsic aggressiveness of the tumor.

## Figures and Tables

**Table 1:** Clinicopathological characteristics of patients stratified according to GFR values at 6 months.

Characteristics	All Patients (*n* = 260)	GFR < 30 (*n* = 44)	GFR = 30–60 (*n* = 77)	GFR ≥ 60 (*n* = 139)	*p* Value
Age (years), (SD)	60.3 (10.6)	60 (8.3)	60.2 (10.1)	60.4 (11.5)	0.973
Gender					0.327
Female	71 (27.3)	8 (18.2)	23 (29.9)	40 (28.8)	
Male	189 (72.7)	36 (81.8)	54 (70.1)	99 (71.2)	
BMI (kg/m^2^), IQR	26.6 (3.7)	27.2 (3.9)	26.9 (4.1)	26.3 (3.3)	0.250
Baseline GFR, IQR	66.4 (18.1)	68.8 (15.3)	65.9 (17.9)	66 (19)	0.645
Nephrectomy history					0.909
Present	235 (90.4)	39 (88.6)	70 (90.9)	126 (90.6)	
Histology					0.254
Clear cell	199 (76.5)	32 (72.7)	55 (71.4)	112 (80.6)	
Non-clear cell	61 (23.5)	12 (27.3)	22 (28.6)	27 (19.4)	
Tumor grade					0.413
I–II	89 (34.2)	14 (31.8)	31 (40.3)	44 (31.7)	
III–IV	171 (65.8)	30 (68.2)	46 (59.7)	95 (68.3)	
Metastatic region sites					
Lung	190 (73.1)	34 (77.3)	48 (62.3)	108 (77.7)	0.040
Liver	51 (19.6)	8 (18.2)	14 (18.2)	29 (20.9)	0.863
Bone	72 (27.7)	8 (18.2)	17 (22.1)	47 (33.8)	0.055
Brain	18 (6.9)	1 (2.3)	8 (10.4)	9 (6.5)	0.228
IMDC					0.014
Favorable	35 (13.5)	9 (20.5)	14 (18.2)	12 (8.6)	
Intermediate	150 (57.7)	28 (63.6)	46 (59.7)	76 (54.7)	
Poor	75 (28.8)	7 (15.9)	17 (22.1)	51 (36.7)	
First-line treatment					0.410
Pazopanib	141 (54.2)	28 (63.6)	42 (54.5)	71 (51.2)	
Sunitinib	93 (35.7)	14 (31.9)	29 (37.7)	50 (35.9)	
Cabozantinib	26 (10.1)	2 (4.5)	6 (7.8)	18 (12.9)	
Second-line treatment					0.948
Nivolumab	81 (48.2)	13 (48.1)	24 (48)	44 (48.4)	
Axitinib	42 (25)	7 (25.9)	14 (28)	21 (23.1)	
Everolimus	38 (22.6)	6 (22.2)	9 (18)	23 (25.3)	
Cabozantinib	7 (4.2)	1 (3.8)	3 (6)	3 (3.2)	
Third-line treatment					0.690
Nivolumab	16 (19.8)	2 (14.3)	6 (23.1)	8 (19.5)	
Axitinib	48 (59.3)	9 (64.3)	15 (57.7)	24(58.5)	
Everolimus	13 (16)	1(7.1)	4(15.4)	8(19.5)	
Cabozantinib	4 (4.9)	2(14.3)	1(3.8)	1(2.4)	

Categorical variables are presented as frequencies and percentages. Abbreviations; IQR: interquartile range; GFR: glomerular filtration rate; BMI: body mass index; IMDC: International Metastatic Renal Cell Carcinoma Database Consortium; SD: standard deviation.

**Table 2:** Univariate Cox regression analysis determining the associations of clinical and pathological variables with PFS and OS.

	PFS		OS	
Variable	HR (95 CI%)	*p* Value	HR (95 CI%)	*p* Value
Age, years	1 (0.98–1.01)	0.910	1 (0.98–1.01)	0.773
Gender (male vs. female)	0.79 (0.58–1.08)	0.149	0.90 (0.64–1.27)	0.563
Histology (non-clear vs. clear)	1.14 (0.82–1.57)	0.413	1.27 (0.88–1.82)	0.195
Tumor grade (III–IV vs. I–II)	0.93 (0.70–1.25)	0.673	1.45 (1.03–2.02)	0.030
Lung metastasis (present vs. absent)	0.78 (0.57–1.07)	0.126	1.08 (0.76–1.54)	0.640
Liver metastasis (present vs. absent)	1.39 (0.98–1.97)	0.062	1.47 (1.01–2.13)	0.043
Bone metastasis (present vs. absent)	1.32 (0.98–1.78)	0.063	1.66 (1.19–2.32)	0.004
Brain metastasis (present vs. absent)	1.18 (0.70–1.99)	0.513	1.88 (1.10–3.22)	0.020
IMDC risk scoring system		<0.001		<0.001
Favorable	1 (reference)		1 (reference)	
Intermediate	1.70 (1.08–2.68)	0.022	2.38 (1.29–4.40)	0.005
Poor	4.36 (2.64–7.20)	<0.001	6.32 (3.32–12.03)	<0.001
GFR at 6 months		0.012		<0.001
≥60	1 (reference)		1 (reference)	
30–60	1.29 (0.83–2.00)	0.258	2.45 (1.29–4.66)	0.006
<30	1.74 (1.16–2.62)	0.007	4.79 (2.62–8.73)	<0.001

Abbreviations; CI: confidence interval; GFR: glomerular filtration rate; HR: hazard ratio; IMDC: International Metastatic Renal Cell Carcinoma Database Consortium; PFS: progression-free survival; OS: overall survival.

**Table 3:** Multivariate Cox regression analysis determining independent predictors of PFS and OS.

	HR	95% CI		*p* Value
		Lower	Upper	
PFS				
GFR at 6 months				0.034
≥60	1 (reference)			
30–60	1.08	0.68	1.72	0.723
<30	1.54	1.01	2.33	0.040
Lung metastasis (present vs. absent)	0.78	0.56	1.09	0.148
Liver metastasis (present vs. absent)	1.32	0.93	1.89	0.116
Bone metastasis (present vs. absent)	1.17	0.86	1.59	0.306
IMDC risk scoring system				<0.001
Favorable	1 (reference)			
Intermediate	1.67	1.05	2.66	0.028
Poor	4.13	2.47	6.91	<0.001
OS				
GFR at 6 months				<0.001
≥60	1 (reference)			
30–60	2.07	1.08	3.98	0.028
<30	3.80	2.06	7.01	<0.001
Liver metastasis (present vs. absent)	1.60	1.09	2.37	0.017
Bone metastasis (present vs. absent)	1.54	1.09	2.18	0.014
Brain metastasis (present vs. absent)	2.44	1.38	4.33	0.002
Histology (non-clear vs. clear cell)	1.34	0.92	1.97	0.124
Tumor grade (III–IV vs. I–II)	1.05	0.74	1.50	0.757
IMDC risk scoring system				<0.001
Favorable	1 (reference)			
Intermediate	2.13	1.16	3.90	0.014
Poor	5.81	3.07	10.99	<0.001

Abbreviations; CI: confidence interval; GFR: glomerular filtration rate; HR: hazard ratio; IMDC: International Metastatic Renal Cell Carcinoma Database Consortium; PFS: progression-free survival; OS: overall survival.

## Data Availability

Data are available upon reasonable request.

## Abbreviations

The following abbreviations are used in this manuscript:

BMI	body mass index
CI	confidence interval
CKD	chronic kidney disease
GFR	glomerular filtration rate
ICIs	immune checkpoint inhibitors
HR	hazard ratio
IMDC	International Metastatic Renal Cell Carcinoma Database Consortium
OS	overall survival
PFS	progression-free survival
mRCC	metastatic renal cell carcinoma
SD	standard deviation
TMA	thrombotic microangiopathy
VEGFR	vascular endothelial growth factor receptor
